# Complete genome sequence of *Pseudomonas fulva* strain MTT5 isolated from the maize (*Zea mays*) phyllosphere

**DOI:** 10.1128/mra.00543-25

**Published:** 2025-09-15

**Authors:** Bashir A. Akhoon, Katie Christensen, Hanna Lefevers, Kendall R. Corbin

**Affiliations:** 1 Department of Horticulture, Martin-Gatton College of Agriculture, Food and Environment, University of Kentucky 4530, Lexington, Kentucky, USA; Loyola University Chicago, Chicago, Illinois, USA

**Keywords:** whole genome, *Pseudomonas*, phyllosphere, maize, Oxford Nanopore Technology, biosynthetic gene clusters, antibiotic

## Abstract

*Pseudomonas fulva* MTT5, isolated from maize leaf surfaces, exhibits broad-spectrum antimicrobial activity. We present the complete genome sequence of *P. fulva* MTT5. The 4.9 Mb genome contains 4,488 predicted genes and a GC content of 61.5%. Identified biosynthetic gene clusters highlight the strain’s potential as a source of novel antibiotics.

## ANNOUNCEMENT

The maize (*Zea mays*) phyllosphere harbors a diverse microbial community with underexplored potential for natural product biosynthesis (1). In our previous study, 237 bacteria were isolated from maize leaves (2). The isolate MTT5 exhibited broad-spectrum antibacterial activity against safe relatives of ESKAPE pathogens, suggesting its potential as a source of antibiotics. Preliminary 16S rRNA gene analysis identified isolate MTT5 as *Pseudomonas* (accession PV624825). To elucidate the genomic basis of this activity and refine its taxonomic placement, complete genome sequencing of MTT5 was performed.

Isolate MTT5 was collected from the adaxial surface of maize (G14R38-GT) grown at the University of Kentucky North Farm (3). The isolate was collected from a tryptic soy agar plate following incubation at 27°C and serial streaking. For antibiotic assays, the isolate was co-cultured with safe ESKAPE relatives using the patch method at 27°C for 3 days, after which zones of inhibition were recorded. Genomic DNA from MTT5 was extracted using the ZymoBIOMICS 96 MagBead DNA Kit (Zymo Research) with the v14 library prep chemistry kit from Oxford Nanopore Technologies (ONT).

**Fig 1:**
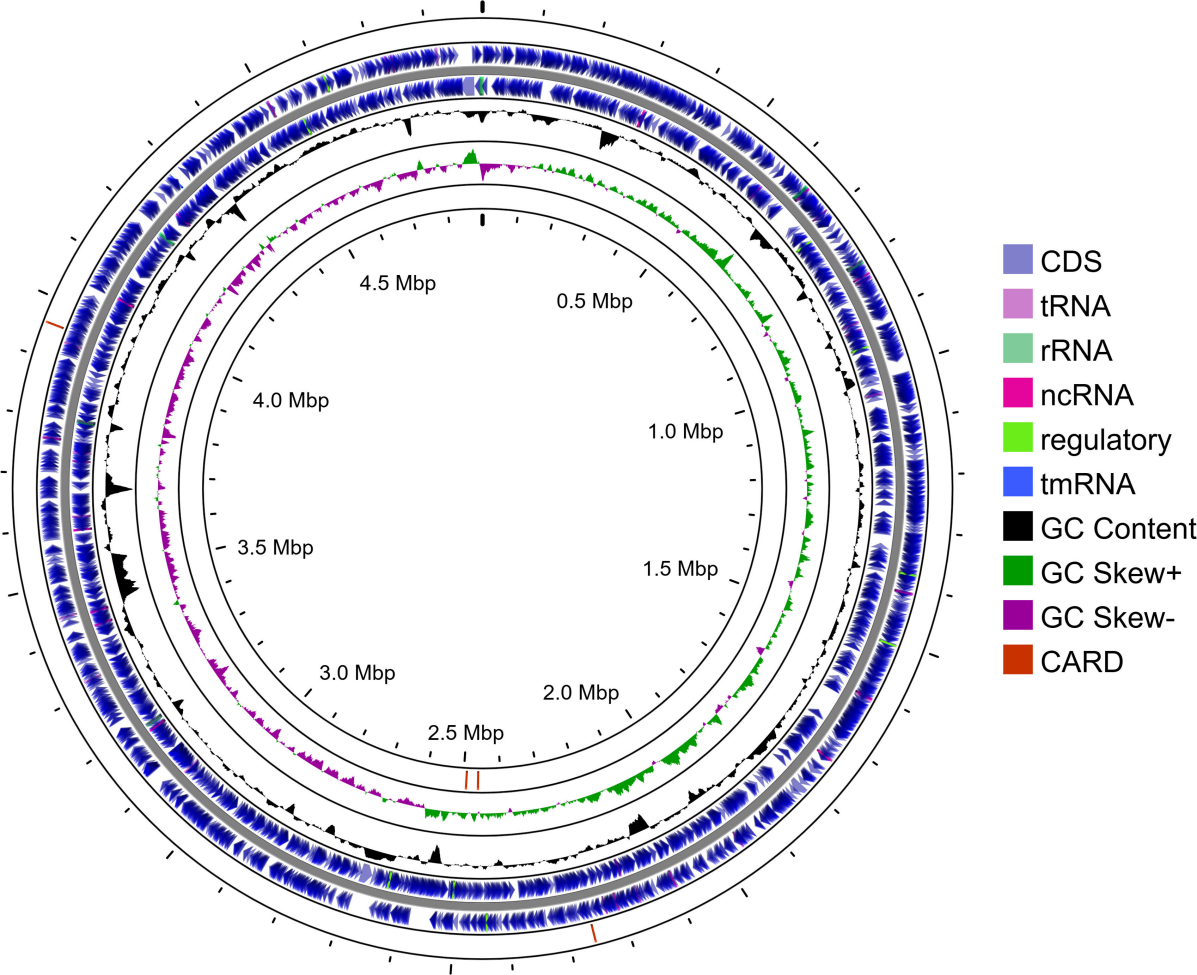
Circular representation of the assembled genome. The outermost and corresponding innermost rings display predicted AMR genes identified using the CARD with the strict cutoff, oriented in the forward and reverse directions, respectively. Inner rings show annotated genomic features, including coding sequences, tRNAs, rRNAs, ncRNAs, regulatory elements, and tmRNAs. The central tracks represent GC content and GC skew (positive in purple, negative in green), highlighting compositional variation across the genome.

Genome sequencing was performed by Plasmidsaurus using the Oxford Nanopore MinION platform. Unless otherwise noted, default parameters were used for all software. Quality control and downsampling were performed using Filtlong v0.2.1. Initially, the bottom 5% of reads were removed using default parameters. The remaining reads were downsampled to 250 Mb to generate a preliminary assembly with Miniasm (release 2024-10-17). Based on the Miniasm output, reads were re-downsampled to ~100× coverage, removing low-quality reads. The final genome assembly was generated using Flye v2.9.1 (https://github.com/mikolmogorov/Flye) and polished post-assembly with Medaka v1.8.0 (https://github.com/nanoporetech/medaka). Assembly quality was assessed using CheckM v1.2.2 (4) and annotation was performed with NCBI Prokaryotic Genome Annotation Pipeline (PGAP) v6.7 (5). Taxonomic classification was refined using Mash v2.3 (6), Sourmash v4.6.1 (7), and CheckM. A total of 260,155 raw reads, totaling ~857 Mbp, with a read N50 of 5.7 kb and maximum read length of 77.7 kb, was generated. The final assembly consisted of a single circular 4,897,385 bp chromosome with 103× assembled coverage, with no plasmids identified. Genome completeness was >99% with negligible contamination (0.1%). A circular genome map was generated using Proksee (8) to visualize genomic features, GC content, GC skew, and predicted antimicrobial resistance (AMR) genes based on the Comprehensive Antibiotic Resistance Database (CARD) (Fig. 1) (9).

4,488 genes were annotated by NCBI PGAP. Mash analysis showed 99.3% identity to *Pseudomonas fulva* NBRC 16637 (accession NZ_JHYU01000001), confirming the identity of MTT5. Nine biosynthetic gene clusters (BGCs) were identified using antiSMASH v7.1.0 (10). These included clusters with 100% similarity to known antimicrobial pathways, such as a hydrogen cyanide biosynthetic cluster, a carotenoid-like terpene cluster, and a nonribosomal peptide synthetase (NRPS) cluster associated with the production of stechlisins, a class of cyclic depsipeptides with antimicrobial activity. Additional BGCs included arylpolyene (40% similarity to APE Vf), redox cofactor-like compounds (13% similarity to lankacidin C), ribosomally synthesized and post-translationally modified peptides (RiPP-like), N-acetylglutaminylglutamine amide, and NRPS-metallophore-associated clusters. These findings substantiate earlier biochemical observations (2) and highlight the potential of *P. fulva* MTT5 as a source of antibiotics and novel secondary metabolites.

## Data Availability

The Whole Genome Shotgun project has been deposited in DDBJ/ENA/GenBank under the accession no. CP191382. The version described in this paper is the first version, CP191382.1. The associated BioProject, BioSample, and raw sequencing data are available under the following accessions: BioProject PRJNA1262510, BioSample SAMN48457711, and SRA SRR33590245. The annotated genome corresponds to *Pseudomonas fulva* strain MTT5.
